# Trading on Food Quality due to Changes in Prices: Are There Any Nutritional Effects?

**DOI:** 10.3390/nu12010023

**Published:** 2019-12-20

**Authors:** Cesar Revoredo-Giha, Faical Akaichi, Neil Chalmers

**Affiliations:** 1 Food Marketing Research, Rural Economy, Environment and Society Department, Scotland’s Rural College, West Mains Road, Edinburgh EH9 3JG, UK; faical.akaichi@sruc.ac.uk; 2 Rowett Institute of Health and Nutrition, University of Aberdeen, Ashgrove Rd W, Aberdeen AB25 2ZD, UK; neil.chalmers@abdn.ac.uk

**Keywords:** food choice, changes in quality, changes in prices, nutrition, United Kingdom, Brexit

## Abstract

The analysis of changes in prices is not only important because they directly affect households’ affordability and, therefore, their food security but also because they may trigger changes in the composition of their food and drink choices. Thus, an increase in prices may force a household with limited resources to choose a bundle of goods with lower prices that substitute their original choices and are probably of lower quality. This paper considers the situation of each UK country and the implications that trading down in quality within a food and drink category has on nutrition. Two motivations to pursue these analyses are to explore the sort of substitutions that households do within a category due to an increase in prices and, in the UK leaving the European Union (Brexit) context, the impact that an increase in food prices may have on nutrition. After computing estimates for trading down for each country for the period 2007–2014, we regress the annual rate of change by nutrient with respect to the annual trading in quality for six food qualities that are major contributors of fat, sugar and salt to the diet. The results indicate that trading down in quality occurs in most of the studied categories and countries, and when households trade down, they move to products with worse nutritional quality. This points out the need to keep improving the quality of products through reformulation, ensure that consumers are well informed of nutritional quality of products and monitor the effect of changes in prices.

## 1. Introduction

Product quality can be described as a bundle of characteristics (attributes) that determine a product’s performance. Major categories of food product quality attributes include food safety (e.g., levels of microbial pathogens and residues), nutritional, value (e.g., compositional integrity and taste), package and process (e.g., animal welfare and environmental impact) attributes [1].

Changes in food prices have the possibility to modify not only the quantity consumed of a particular product but also the quality of the products that are chosen, for instance, within a food category [2,3].

The United Kingdom (UK) Department of Food, Environment and Rural Affairs (Defra) produces, as part of its regular publication “Family Food” [4,5], a calculation of the changes in quality due to changes in prices (i.e., trading up/down in quality). In a study considering the years 2007 to 2014, it showed that when considering several food and drink categories, trading down in quality was a very common pattern in UK households together and first income decile households. In fact, their calculation showed that households traded down significantly in 15 out of 22 food and drink categories in the case of all the UK households and 14 out of 22 food and drink categories for the poorest income decile.

It is important to note that Defra’s ‘trading down in quality’ definition considers that it “means switching to purchases of cheaper products within a food grouping [i.e., a category]. Cheaper is equivalent to lower quality in some way. The quality reduction could occur in any quality attribute of the product, e.g., packaging, brand name, provenance, nutrient content, taste. “Trading down into a completely different food type is not captured” [4,5]. This, however, does not need to be the case as it depends on the attributes behind the idea of quality. For instance, if cheaper products are less tasteful than the more expensive ones but they are also healthier due to a better nutritional composition (e.g., lower sugar content), then consumers probably are not worse off by the substitution from a more expensive product to a cheaper one. In other terms, the relationship between trading down in quality and nutrition is an empirical question, as the change in the composition of the basket might not have any nutritional implication.

The contribution of this paper and its aim is to explore what the implications of increases in food and drink prices are in terms of nutrition; more specifically, when consumers trade-down in quality, do they choose products that are worse off in nutritional quality? Moreover, the analysis of this question is done considering the four UK countries (i.e., England, Northern Ireland, Scotland and Wales), which may have different reactions to changes in prices.

There are several reasons for exploring the aforementioned question; one is to explore the effect of changes in purchases made after recession, which has radically changed consumers’ purchase behaviour to the point that the new market situation has been described as “age of thrift” [6]. Another reason to explore the nutritional effect of price increases is that it has become a constant concern in the context of the UK leaving the European Union (Brexit), e.g., due to a depreciation of the British Pound (GBP) or new trading conditions imply higher import costs for the country. Thus, potential price rises due to Brexit have been mentioned by several sources [7,8,9]. Johnson et al. [10] pointed out that the Bank of England have estimated that 60 per cent of the fall in the GBP was passed through higher prices to firms and consumers and that a higher percentage was expected in the long term. In this context, where healthy foods have been portrayed as relatively expensive [11,12] and the UK self-sufficiency rate is 58 per cent in vegetables and 11 per cent in fruit-price increases will certainly have an impact on consumers’ healthy diet.

As mentioned, Defra’s calculations of trading in quality were done for all the UK households and those on the first income decile; however, the availability of disaggregated data behind the Family Food publication from the UK Data Archive allows extending Defra’s analysis by carrying it out by UK country (i.e., England, Northern Ireland, Scotland and Wales). These additional computations are interesting in the Brexit context given the potential differences by country.

The structure of the paper is as follows: it starts with a literature review that briefly summarises the literature of consumers’ quality choice and prices. The next section presents the methodology and the data used for the analysis. It is then followed by the discussion of the results, i.e., the estimation of the trading in quality by UK country and their impact on nutrition. Finally, conclusions from the analysis are stated.

## 2. Literature Review

The analysis of price changes is important not only due to the fact that they directly affect households’ affordability and, therefore, their food security, but also because they may trigger changes in the composition of food and drink choices, which have an impact on the quality of the products they buy.

The discussion of changes in prices and their effect on quality changes has been extensively discussed in the demand analysis literature as regards work done with family budget data related to the estimation of price elasticities [2,13,14,15].

As pointed out by Theil [15], most “commodities” distinguished in budget enquiries are heterogeneous; often they can be described as “aggregates” of more perfectly homogeneous “qualities.” A consequence of this well-known fact is that an increase in the expenditures on a certain commodity brought about by, e.g., an increase in income may be caused partly by an increase in the quantity bought (measured in physical units), partly by a transition to a better quality.

In household surveys reporting both expenditures and physical quantities, it is possible to divide one by the other to obtain unit values. These unit values suggest that there is substantial spatial variation in prices; however, it is not possible to use unit values as direct substitutes for true market prices in the analysis of demand patterns. Consumers choose the quality of their purchases, and unit values reflect this choice. Moreover, quality choice may itself reflect the influence of prices as consumers respond to price changes by altering both quantity and quality [2].

McKelvey [3] reported that when estimating demand for a heterogeneous commodity group that is comprised of many different individual goods, commonly used demand system estimation techniques may produce erroneous estimates of the price elasticity of quantity demanded. If quality response to price is ignored, estimated price elasticities of quantity demand will conflate responses on the quantity and quality margins. Policy makers in many countries are considering, or have imposed, taxes to cut intake of unhealthy items like fats and sugar-sweetened beverages. Proponents of taxes assume that quantity demand is fairly responsive to price. The empirical estimates of demand elasticities that applied economists have published over several decades undoubtedly contribute to this view. Yet evidence in many of these countries is from household surveys, with budget shares that vary with the choice of quantity and quality. If quality response to price is ignored, estimated price elasticities of quantity demand include quality responses and overstating likely effects of taxes.

The source of this problem is that it has become increasingly common to use commodity group budget share as the dependent variable when estimating demand systems. When regressing budget share on price, there are two pathways through which price can impact budget share. Again, taking meat as our example, one possibility is that when the price of meat increases, the household might decide to decrease the quantity of meat purchased, thus reducing the budget share devoted to meat. Another possibility is that the household may substitute to cheaper cuts of meat, which would also reduce the budget share devoted to meat. Either way, one would observe a negative correlation between meat price and meat budget share, and this regression provides no way to determine whether this correlation is due to a response on the quantity margin or a response on the quality margin. Moreover, confusing these two responses may result in perverse policy decisions, since taxing a commodity group whose expenditure is responsive to price changes could decrease the quantity consumed or it could instead cause consumers to substitute to lower quality versions of the commodity.

As regards the relationship between prices and nutrition, Huang [16] linked food choice with nutritional status in the context of the classical demand framework by developing a procedure to measure nutrient availability by way of demand elasticities for food items from a traditional demand analysis. Although it is possible to find the relationship between changes in prices and the change in nutrients using Huang’s procedure, it does not provide an aggregated index of quality such as in the Deaton and McKelvey’s method [2,3]. In what remains of the paper, their index will be used to explore whether changes in prices had any impact on the nutritional quality of the diet.

## 3. Materials and Methods

### 3.1. Methods

The starting point of the methodology is based on McKelvey [3], who studied the relationships between unit value and the quality demanded on commodity groups. He follows Deaton [2] in assuming that commodities are divided into groups, each consisting of a number of consumer goods; for instance, if the group is confectionery, it will comprise chocolates, candies. The expenditure for group c is given by:

(1) $$E_{c}=p_{c}\cdot q_{c}$$

where $p_{c}$ and $q_{c}$ are vectors containing the price and quantity of each consumption good in the group. The price vector is then decomposed as:

(2) $$p_{c}={\bar{p}}_{c}\cdot p_{c}^{*}$$

where $p_{c}^{*}$ is a vector capturing the relative price of commodities within the group and ${\bar{p}}_{c}$ is a scalar capturing the component of price that is common to all the commodities in the group (e.g., a Laspeyres price index). In this analysis ${\bar{p}}_{c}$ is understood as a price index for the category c.

Replacing (2) into (1), one obtains:

(3) $$E_{c}={\bar{p}}_{c}\cdot p_{c}^{*}\cdot q_{c}$$

Multiplying and dividing the right hand side by the total quantity purchased, i.e., $Q_{c}=1\cdot q_{c}$ and replacing $v_{c}=\frac{p_{c}^{*}\cdot q_{c}}{Q_{c}}$, (3) becomes:

(4) $$E_{c}={\bar{p}}_{c}\cdot Q_{c}\cdot v_{c}$$

As explained by McKelvey [3], $v_{c}$ is a measure of the quality of group c purchases. If the relative prices are constant, a higher $v_{c}$ means that the chosen $q_{c}$ is more expensive per unit of group c consumption and this is interpreted as buying a higher quality of group c goods. However, note that the term quality is used in vague way, as it is not associated with any particular attribute.

Expressing (4) as discrete changes from period t-1 to t we obtain (5):

(5) $$\left(1+{\hat{E}}_{\mathrm{ct}}\right)=\left(1+{\hat{\overset{↼}{p}}}_{\mathrm{ct}}\right)\cdot \left(1+{\hat{Q}}_{\mathrm{ct}}\right)\cdot \left(1+{\hat{v}}_{\mathrm{ct}}\right)$$

where ‘^’ indicate the relative change with respect to the previous period. From (5), it is clear that to keep the expenditure constant after an increase in the category price, a consumer can reduce its total quantity purchase or trade down the quality of the purchases or a combination of both. If ${\hat{v}}_{\mathrm{ct}}$ is negative, the consumer is trading down in quality.

The next step once ${\hat{v}}_{\mathrm{ct}}$ is computed from (5) is to explore to what extent the change in quality as explained above is associated with a change of nutrients. This is an empirical question, as the cheaper products in a category might not be least nutritional ones. To do this, we considered the following pooled regression based on Suits [17]:

(6) $${\hat{N}}_{\mathrm{ijt}}=\alpha +\alpha_{\mathrm{Eng}}+\alpha_{\mathrm{NI}}+\alpha_{\mathrm{Sc}}+\alpha_{W}+\beta \cdot {\hat{v}}_{\mathrm{ct}}$$

where ${\hat{N}}_{\mathrm{ijt}}$ is the relative change of nutrient i in country j in time period t; α and β are the intercept and slope of the regression, respectively, and $\alpha_{\mathrm{Eng}}$, $\alpha_{\mathrm{NI}}$, $\alpha_{\mathrm{Sc}}$ and $\alpha_{W}$ are parameters based on dummies (i.e., that take the value of one if the observation is for the country and zero otherwise) that measure the deviations with respect to α for the cases of the four UK countries, namely England (Eng), Northern Ireland (NI), Scotland (Sc) and Wales (W).

The most important parameter of regression (6) is the slope β, which indicates the reaction of the nutrient (i.e., how it changes in relative terms) to a trading in quality. Note that if the coefficient of the slope is negative and statistically significant (using a t test to check whether the slope is different than zero), it means that trading down in quality (which is a negative relative change in the real unit value) increases the nutrients. In contrast, if the slope is positive, trading down in quality decreases the demand for nutrients. Of course, if the slope is not statistically significant, it means that trading in quality is unrelated to nutrition.

### 3.2. Data Used on the Analysis

The data available for the computation of the trading up/down in quality are disaggregated information from the Defra’s “Family Food” publication for each of the four UK countries. These data provide information about weekly per capita expenditures and purchases [4,5].

The prices used for the categories were from the Office for National Statistics (ONS) Retail Price Indices for the studied groups (base 1987 = 100), which match the groups of the Family Food statistics.

The information about nutrients per food category was taken from disaggregated data underpinning the “Family Food” publication and can be found in the UK Data Archive as part of the Living Costs and Food Surveys. These nutrient intakes are calculated from food purchases using nutrient composition data supplied by Public Health England (PHE) [4,5]. The majority of the data are from PHE’s nutrient analysis programme, supplemented by values from manufacturers and retailers. The nutrient conversion excludes inedible parts of purchased foods, such as fish heads, banana peels; it assumes all food is eaten. Intakes from dietary supplements are not included in any of the tables. The nutrients categories considered in the analysis were energy (i.e., calories), macronutrients (i.e., fat, carbohydrates and proteins), saturated fats, Non-milk extrinsic (NME) sugars, sodium, total Sugars and cholesterol.

In terms of the categories used for the analysis, these included the following Defra’s Family Food categories. Note that the aggregation of the groups is due to the fact that the retail price indices are only available on an aggregated way:

**Food and drink**: which was made of: milk and milk products excluding cheese, cheese, carcase meat, non-carcase meat and meat products, fish, eggs, fats, sugar and preserves, fresh and processed fruit and vegetables, including potatoes, bread, flour, cakes, buns and pastries, biscuits and crispbreads, other cereals and cereal products, beverages, other food and drink, soft drinks, confectionery and alcoholic drinks.

**Biscuits and cakes**: chocolate biscuits, sweet biscuits (not chocolate) and cereal bars, cream crackers and other unsweetened biscuits and crispbread.

**Confectionery**: chocolate bars—solid, chocolate bars—filled, chewing gum, mints and boiled sweets, fudges, toffees, caramels and takeaway confectionery.

**Processed potato products**: chips—frozen or not frozen, takeaway chips, instant potato, canned potatoes, crisps and potato snacks and other potato products, frozen or not frozen.

**Processed vegetables (excluding processed potatoes)**: tomatoes, canned or bottled, peas, canned, beans canned, baked beans in sauce, other canned beans and pulses, other canned vegetables, dried pulses other than air-dried, air-dried vegetables, vegetable juices and purees, tomato puree and vegetable purees, vegetable juices, e.g., tomato juice, carrot juice, peas, frozen, beans, frozen, ready meals and other vegetable products (including takeaways), ready meals and other vegetable products, frozen or not frozen, all vegetable takeaway products and other frozen vegetables.

**Soft drinks**: concentrated (not low calorie), not concentrated (not low calorie), concentrated (low calorie) and not concentrated (low calorie).

Note that the cases of biscuits and cakes, confectionery and soft drinks are part of the discretionary food categories as defined by the Food Standard Scotland (FSS); these are categories not necessary for nutrition and which are contributors of sugar, saturated fats and salts [18]. Thus, according to the FSS, these foods have a significant impact on the diet, accounting for, on average, about one-fifth of total calories, total fat and saturated fats and over half of daily free sugars consumption. Moreover, the FSS concluded that a key step towards meeting dietary goals would be to reduce the intake of these foods.

## 4. Results and Discussion

This section presents two sets of results from the analysis: first, the trading in quality considering for all the selected categories, the changes in prices between 2007 and 2014 and the reaction in terms of change in expenditure, change in quantities purchases and change in the composition of the purchases (i.e., trading in quality). Second, the results of the regressions between the change in nutrients per year between 2007 and 2014 (i.e., 28 observations, this is seven growth changes multiplied by four UK countries) and the trading in quality. It is important to mention that the reason why this discussion does not compare the results with other results from the UK or elsewhere is due to the fact that to our knowledge of this type of analysis (i.e., the relationship between nutrients and trading down in quality) has not been studied before.

### 4.1. Trading in Quality by Food Category

**Figure 1:**
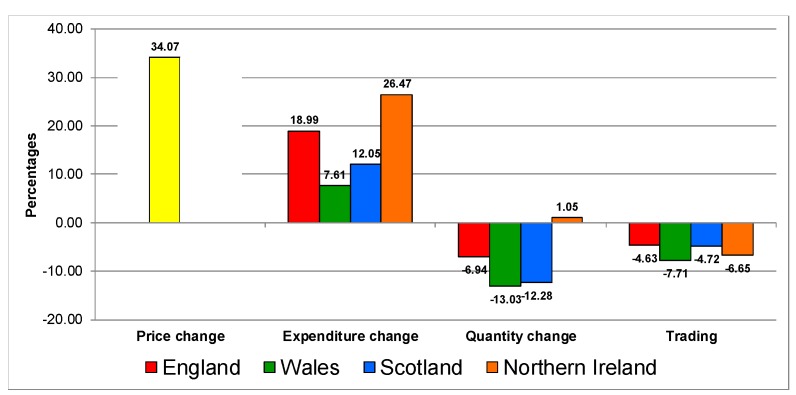
Trading in quality on the food and drink category in UK 2007–2014.

**Figure 2:**
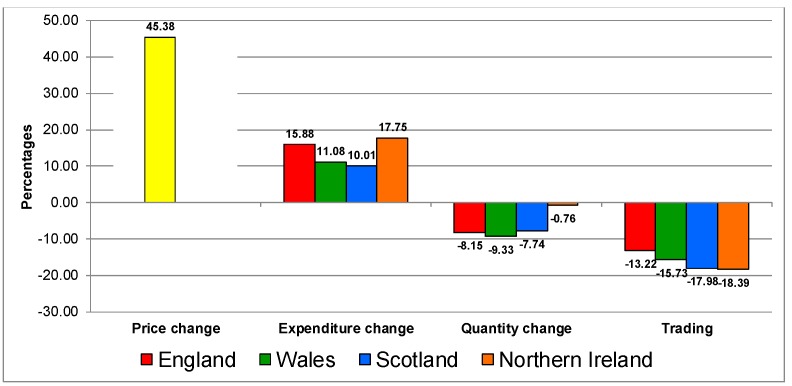
Trading in quality on the biscuits and cake category in UK 2007–2014.

**Figure 3:**
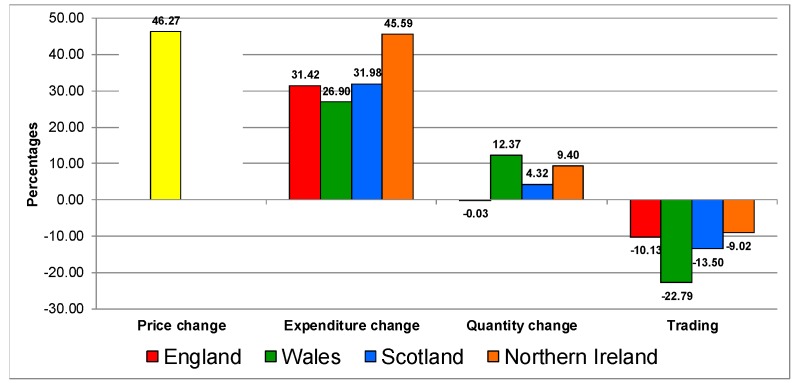
Trading in quality on the confectionery category in UK 2007–2014.

**Figure 4:**
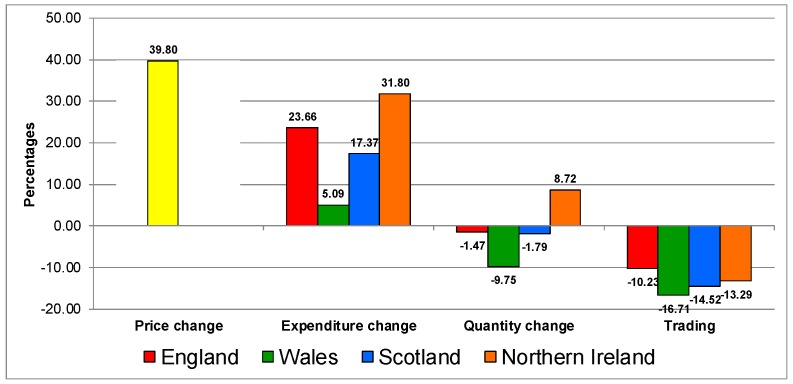
Trading in quality on the processed potatoes category in UK 2007–2014.

**Figure 5:**
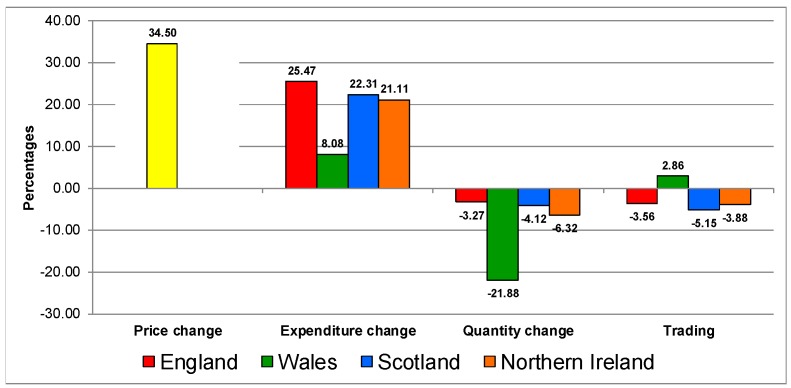
Trading in quality on the processed vegetables category in UK 2007–2014.

**Figure 6:**
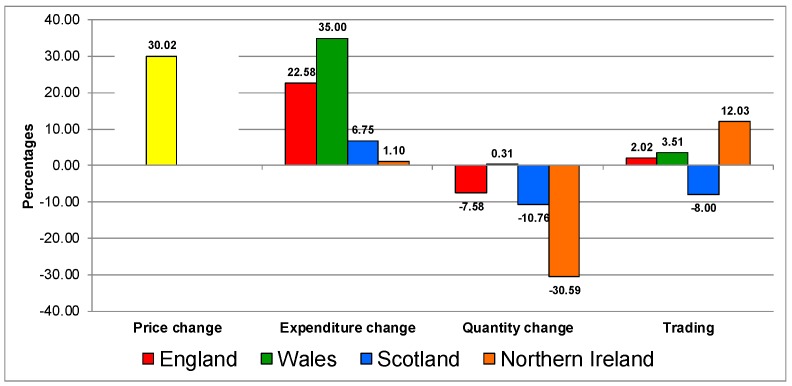
Trading in quality on the soft drinks category in UK 2007–2014.

Figure 1, Figure 2, Figure 3, Figure 4, Figure 5 and Figure 6 present the trading in quality analyses by UK country by category. For the aggregated food and drink category (Figure 1), the reaction to the increase in prices is very similar in all countries. It increases in expenditure, decreases in quantities (except in Northern Ireland) and all of them show trading down in quality.

Figure 2 shows the response for biscuits and cakes category. The response to the increase in prices on the category was similar in all the countries, characterised by increases in expenditure, decreases in quantities and by trading down in quality.

Figure 3 shows the results for the confectionery category, which is similar to the biscuits and cakes category. The response to the increase in prices on the category was similar in all countries, characterised by increases in expenditure, decreases in quantities purchases (England’s quantity remained the same) and all countries trading down in quality.

The increase in the price of processed potatoes (Figure 4) was followed by an increase in expenditure, decrease in quantity in all countries except in Northern Ireland and trading down in all the UK countries.

Figure 5 shows that increases in prices of processed vegetables was followed up on average by an increase in expenditure, a decrease in quantity (particularly in Wales) and trading down in most countries except in Wales.

The results to an increase in the price of soft drinks was more varied (Figure 6). It showed an increase in in expenditure; quantities decreased in all countries except Wales, which remained almost the same. All countries except Scotland traded up in quality.

### 4.2. Nutritional Effect of Trading in Quality

Given the prevalence of trading down in quality in all the UK countries and in all the studied food and drink categories, it is important to analyse whether by purchasing cheaper products within a category, households are moving to a poorer nutritional quality basket—this is done in the next section.

This section presents the results of the regressions between nutrients and trading in quality. The selected nutritional items were energy (calories); macronutrients: fat, carbohydrates and proteins; nutrients that are harmful in excess: saturated fatty acids, non-milk extrinsic sugars and sodium; total sugars and cholesterol.

The relative change of each nutrient in each country was regressed with respect to the relative trading in quality. Since both the dependent and independent variable are in relative change over time, the coefficient can be interpreted as an elasticity (i.e., the reaction of the quantity consumed of a particular nutrient due to a change in the category trading in quality index).

Table 1, Table 2, Table 3, Table 4, Table 5 and Table 6 present the results for all the studied food and drink categories. Note that each line of the Tables is one regression, i.e., each nutrient with respect to the trading in quality. In addition, recall that the most important coefficient is β, which indicates the percentage change in a nutrient due to a percentage change in trade in quality index. If the value of β is negative, it means that trading down in quality generates an increase in the nutrient consumption.

As shown in Table 1, the UK country dummies representing the deviations to the mean coefficients were not statistically significant. However, the value of β was statistically significant in all the regressions fluctuating from −0.7 (sodium) to −1.37 (NME sugars). The results indicate that trading down in quality increases all the nutrients on the food and drink categories. On the negative side, the results show that increases in the prices of the category encourage consumers to reduce their outlay by substituting within the category towards the uptake of products that are higher in saturated fats, sugar and salt.

The above results are of importance because the latest information National Diet and Nutrition Survey (NDNS) [19] collected from 2014 to 2016 show that sugar makes up 13.5 per cent of 4–10 year-olds and 14.1% of 11–18 year-olds daily calorie intake respectively when the official recommendation is to limit sugar to no more than 5%. Moreover, the survey confirms the UK population continues to consume too much saturated fat and not enough fruit, vegetables and fibre. Thus, the average saturated fat intake for adults (i.e., 19- to 64-year-olds) is 12.5 per cent of daily calorie intake, above the 11 per cent recommended maximum. The results from Table 1 indicate that an increase in food and drink prices may bring product substitutions within the food and drink category that may deteriorate even more the nutritional situation of the UK population.

As none of the remaining regressions showed that the UK country dummies were significant, they were eliminated from the remaining regressions. Table 2 presents the regressions for biscuits and cakes. The impact of trading down indicates that consumers substitute for products that are cheaper and higher in NME sugar and saturated fat. This result is important because biscuits and cakes are classified by the FSS [18] as a discretionary category and are top contributors of saturated fat and sugar on the diet. Therefore, if the price of the category increases (as shown in Figure 2), consumers would substitute towards products within the category of poorer nutritional quality going against the FSS [18] recommendations to meet dietary goals.

Note that a potential solution to avoid the worsening of nutritional quality is through reformulation of the products within a category, so that when consumers make their substitutions, their nutrition does not suffer. However, it is important to highlight that the biscuits and cakes category is one where the goals of the PHE reformulation programme (which is focused on sugar and calories) is not being achieved [20]. The information to date indicates that the sugar content has not reached the 5 per cent reduction set for 2017—in fact the average sugar of the category has not changed at all, which means that the goal of 20 per cent reduction for 2020 is highly unlikely to be reached.

Table 3 shows that in the case of confectionery, trading down has an impact on calories and carbohydrates but particularly important is that it increases sugars (particularly NME sugars, with the highest β coefficient equal to −0.52). This is particularly important because confectionery is the greatest contributor of sugar on the diet and another discretionary food category [18].

According to information from PHE [20], similar to the case of biscuits and cakes, the goal of sugar reduction for 2017 was not achieved (i.e., chocolate confectionery content of sugar did not change at all and in the case of sugar confectionery, it decreased by 1 per cent).

Table 4 present the case of processed potatoes. This is a significant category because some of its products (e.g., chips) are associated with takeaway food in the UK. Moreover, as shown by Defra’s Family Food, chips are still the UK’s preferred form of potato [21].

As shown in the Table, no significant relationship was found between trading down/up on processed potatoes purchases and the nutrients. It is important to remember that these results do not indicate that processed potatoes, such as chips, do not contribute, for instance, with, e.g., fat to the diet; they indicate that trading in quality does not have nutritional effects. 

Table 5 presents the results for processed vegetables. As shown above this is a heterogeneous category with several subcategories known for the presence of NME sugar (e.g., baked beans) or fat (e.g., takeaways).

The regressions show that trading down in quality has a significant impact on energy, fat, fatty acids and NME sugars. This may indicate the need to monitoring the category for the so called “ultraprocessed” processed food, which are typically energy dense; have a high glycaemic load; are low in dietary fibre, micronutrients and phytochemicals; and are high in unhealthy types of dietary fat, free sugars and sodium [22].

Table 6 shows the regressions for soft drinks. Similar to the case of processed potatoes, no statistically significant relationship was found between trading in quality in soft drink purchases and the nutrients. As shown in Figure 6, only Scotland traded down in this category; however, the results indicated that the change was not significant in terms of change in NME sugar, which is the most important nutritional issue within the category.

Recent information from the UK Food and Drink Federation [23] indicates that soft drinks included within the Soft Drinks Industry Levy had reduced sugars by 11 per cent and calories in single serve products by 6 per cent. Moreover, even before coming into effect, the levy was already working as about 50 per cent of manufacturers have reformulated their drinks. Although announce in March 2016, the levy started to operate in April 2018 and companies are supposed to pay 24 pennies per litre of drink if it contains 8 g of sugar per 100 millilitres or 18 pennies per litre of drink if it contains between 5–8 g of sugar per 100 millilitres.

## 5. Conclusions

The UK Department of Environment, Food and Rural Affairs (Defra) publishes an estimation of trading in quality; i.e., the change in the composition of the food basket or in a particular category when the food prices or the prices of a category changes. When consumers trade down in quality, it means that they choose a cheaper basket, which is assumed to be of poorer quality.

The term quality in the aforementioned context is very vague as attributes behind quality, e.g., flavour and colour, are not mentioned. Therefore, the contribution of this paper has been to consider the degree of association between trading down in quality and nutrition (i.e., we selected nutrition as the quality attribute). This, of course, is an empirical question, as the change in the composition of the basket might not have any nutritional implication.

The topic is important because changes in prices may force consumers to not only change the quantities consumed but also the composition of their basket, which may worsen nutritional constraints. The analysis was carried out for six food and drink categories and for pooled data of all the UK countries.

The results indicate that changes in category prices affect not only expenditures and the quantities purchased but also the choice of products within a category, which have implications for the quality of the diet.

In general, UK countries responded similarly to increases in prices (though there were differences in some categories). Trading down in quality was a common reaction to the increase in prices in all countries. In addition, it was found that trading down in quality, i.e., substituting toward cheaper products within a category, depending on the category, was found to be associated with increases in energy, fats, fatty acids, NME sugars and carbohydrates.

Trading down in quality in cakes, biscuits and confectionary was found to increase NME sugars and fat in the diet. Moreover, in the case of processed vegetable products, trading down in quality triggers an increase in foods higher in energy, fats, fatty acids and NME sugars. All these products enter into the description of ultra-processed products and should be monitored to avoid detrimental effects on the diet.

Given the results of the analysis, there is a clear need to not only monitor food prices and ensure that consumers understand the nutritional information in labels, as households might worsen their consumption when they choose cheaper products, but also from the supply side, to continue with the reformulation campaign, as it will stop consumers worsening their diets when they make substitutions. These results are important in the context of exiting the European Union, because this means that if prices increase due to a depreciation of the UK sterling pound, different tariffs, or higher international prices, then experiencing negative changes in nutritional quality will be possible. 

A caveat of the analysis is its aggregated nature. Nevertheless, the results are not unreasonable, and they are statistically significant. Further work should consider more disaggregated datasets, i.e., at the level of households, for longer time spans, and explore the substitutions that consumers make. This would require the construction of price indices at a more disaggregated level.

## Figures and Tables

**Table 1:** Food and drink—regressions between nutrients and trading in quality.

Nutrient	Regression Coefficients						Intercept Dummies by Country												R^2^
	α	*t*-Stat.	Sig	β	*t*-Stat.	Sig	England	*t*-Stat.	Sig	Wales	*t*-Stat.	Sig	Scotland	*t*-Stat.	Sig	*n*. Ireland	*t*-stat.	Sig	
Energy	−1.82	−2.58	*	−0.90	−4.81	*	0.37	0.46		−1.17	−0.99		−0.11	−0.07		0.91	0.78		0.37
Fat	−1.82	−2.31	*	−0.93	−4.70	*	0.55	0.55		−1.40	−1.08		−0.17	−0.11		1.02	0.63		0.32
Carbohydrates	−1.81	−2.36	*	−0.84	−4.28	*	0.30	0.37		−0.95	−0.75		−0.06	−0.03		0.71	0.70		0.31
Proteins	−1.81	−3.06	*	−0.71	−2.82	*	0.42	0.54		−1.18	−0.84		−0.42	−0.37		1.18	1.09		0.30
Saturated	−2.13	−2.74	*	−1.04	−5.15	*	0.43	0.47		−1.17	−0.92		−0.38	−0.26		1.11	0.75		0.39
NME Sugars	−2.51	−2.07		−1.37	−3.82	*	−0.01	−0.01		0.03	0.01		−0.43	−0.21		0.41	0.19		0.30
Sodium	−1.91	−2.70	*	−0.70	−4.36	*	0.53	0.61		−1.17	−1.06		0.07	0.04		0.57	0.50		0.26
Total Sugars	−2.17	−2.16	*	−1.09	−4.25	*	0.05	0.04		−0.29	−0.17		−0.50	−0.28		0.74	0.40		0.30
Cholesterol	−2.58	−3.33	*	−1.00	−3.21	*	0.46	0.43		−1.25	−0.69		−0.43	−0.28		1.22	0.95		0.32

Note: * stands for statistically significant at 95 per cent. Each regression was run with 28 observations.

**Table 2:** Biscuits and cakes—regressions between nutrients and trading in quality.

Nutrient	Regression Coefficients						R^2^
	α	*t*-stat.	Sig	β	*t*-Stat.	Sig	
Energy	−1.85	−2.18	*	−0.33	−1.88		0.17
Fat	−1.70	−1.54		−0.27	−1.46		0.10
Carbohydrates	−1.90	−2.32	*	−0.33	−1.90		0.18
Proteins	−1.93	−3.27	*	−0.30	−2.02		0.17
Saturated	−2.21	−2.53	*	−0.40	−2.77	*	0.20
NME Sugars	−3.15	−3.10	*	−0.75	−3.70	*	0.35
Sodium	−1.96	−2.43	*	−0.27	−2.01		0.13
Total Sugars	−2.56	−2.98	*	−0.55	−3.14	*	0.28
Cholesterol	−2.55	−3.02	*	−0.34	−1.66		0.13

Note: * stands for statistically significant at 95 per cent significant. Each regression was run with 28 observations.

**Table 3:** Confectionery—regressions between nutrients and trading in quality.

Nutrient	Regression Coefficients						R^2^
	α	*t*-Stat.	Sig	β	*t*-Stat.	Sig	
Energy	−1.52	−1.68		−0.23	−2.12	*	0.08
Fat	−1.53	−1.59		−0.24	−1.98		0.07
Carbohydrates	−1.62	−1.75		−0.26	−2.52	*	0.10
Proteins	−1.53	−1.90		−0.15	−1.65		0.04
Saturated	−1.75	−1.87		−0.24	−1.75		0.07
NME Sugars	−2.40	−1.78		−0.52	−2.74	*	0.16
Sodium	−1.82	−2.18	*	−0.25	−2.67	*	0.11
Total Sugars	−2.07	−1.93		−0.41	−2.57	*	0.15
Cholesterol	−2.03	−1.90		−0.14	−0.93		0.02

Note: * stands for statistically significant at 95 per cent significant. Each regression was run with 28 observations.

**Table 4:** Processed potatoes—regressions between nutrients and trading in quality.

Nutrient	Regression Coefficients						R^2^
	α	*t*-Stat.	Sig	β	*t*-Stat.	Sig	
Energy	−1.33	−1.39		−0.14	−0.80		0.05
Fat	−1.35	−1.35		−0.16	−0.94		0.05
Carbohydrates	−1.31	−1.35		−0.11	−0.63		0.03
Proteins	−1.40	−1.69		−0.09	−0.53		0.03
Saturated	−1.57	−1.51		−0.16	−0.94		0.06
NME Sugars	−1.63	−0.96		−0.14	−0.49		0.02
Sodium	−1.50	−1.76		−0.09	−0.67		0.02
Total Sugars	−1.41	−1.02		−0.08	−0.35		0.01
Cholesterol	−2.07	−1.93		−0.17	−0.77		0.06

**Table 5:** Processed vegetables—regressions between nutrients and trading in quality.

Nutrient	Regression Coefficients						R^2^
	α	*t*-Stat.	Sig	β	*t*-Stat.	Sig	
Energy	−1.13	−1.44		−0.30	−2.09	*	0.15
Fat	−1.13	−1.29		−0.37	−2.65	*	0.18
Carbohydrates	−1.16	−1.39		−0.21	−1.45		0.07
Proteins	−1.28	−1.84		−0.30	−2.05		0.17
Saturated	−1.37	−1.78		−0.52	−3.53	*	0.34
NME Sugars	−1.48	−1.16		−0.53	−2.33	*	0.17
Sodium	−1.40	−1.97		−0.34	−2.91	*	0.21
Total Sugars	−1.35	−1.32		−0.44	−2.27	*	0.18
Cholesterol	−1.81	−1.89		−0.30	−1.44		0.10

Note: * stands for statistically significant at 95 per cent significant. Each regression was run with 28 observations.

**Table 6:** Soft drinks—regressions between nutrients and trading in quality.

Nutrient	Regression Coefficients						R^2^
	α	*t*-Stat.	Sig	β	*t*-Stat.	Sig	
Energy	−1.07	−1.23		−0.02	−0.13		0.00
Fat	−1.01	−1.01		−0.08	−0.40		0.01
Carbohydrates	−1.09	−1.27		−0.04	−0.26		0.00
Proteins	−1.25	−1.62		0.04	0.33		0.00
Saturated	−1.30	−1.33		0.05	0.26		0.00
NME Sugars	−1.45	−1.05		0.15	0.58		0.02
Sodium	−1.33	−1.66		−0.01	−0.07		0.00
Total Sugars	−1.31	−1.18		0.08	0.40		0.01
Cholesterol	−1.73	−1.69		−0.05	−0.29		0.00
